# Pelvic Lymphadenectomy in the Treatment of Invasive Bladder Cancer: Literature Review

**DOI:** 10.1155/2011/701481

**Published:** 2011-08-29

**Authors:** Ehab A. Elzayat, Ali A. Al-Zahrani

**Affiliations:** ^1^Department of Urology, Al-Azhar University, Cairo, Egypt; ^2^Department of Urology, University of Dammam, Saudi Arabia

## Abstract

The standard surgical treatment of invasive bladder cancer is the radical cystectomy and pelvic lymph node dissection (PLND). Up to one-third of patients with invasive bladder cancer have lymph node metastasis. Thus, PLND has important therapeutic and prognostic benefits. The number of lymph nodes that should be removed and the extent of the PLND are still a controversial issue. Recently, the trend of PLND increased toward more extended PLND. Several prognostic factors related to PLND were reported in the literature. In this paper, we will discuss the different PLND templates, number of lymph nodes that should be resected, lymph node density, lymphovascular invasion, tumor burden, extracapsular extension, and the aggregate lymph node metastasis diameter.

## 1. Introduction

According to cancer statistic 2010, bladder cancer is the fourth most common tumor in men in the United States, the number of new cases diagnosed were estimated to be 70,530 (52,760 men and 17,770 women), leading to 14,680 deaths [1]. In Europe, bladder cancer represents 6.6% and 2.1% of the total cancers and 4.1 % and 1.8% of total deaths for cancer in men and women, respectively [2].

Radical cystectomy (RC) accompanied by pelvic lymph node dissection (PLND) is still the gold standard surgical treatment for muscle invasive bladder cancer. In the past, the role of PLND was purely diagnostic to rule out lymph nodes (LN) metastasis; currently, PLND is considered an essential part of surgical treatment of bladder cancer. Fourteen to 30% of the patients of invasive bladder cancer have LN metastasis at the time of RC [3, 4]. The incidence of LN metastasis increased with higher tumor stage. LN-positive disease was found in 5% patients with superficial bladder tumors and 18% of patients with P2 tumor, 26% of patients with P3a disease, and 46% of patients with P3b, and 42% of patients with P4 tumors [3].

It is common knowledge that the patients with LN metastasis have a poor prognosis, Vieweg et al. [5] reported on 140 patients with LN positive, 25.7% were disease-free, and 15.7% surviving beyond 5 years. In another study, the 3-year survival in patients who underwent RC and PLND with negative and positive LN was 78.3% and 37.8%, respectively [6]. Thus, PLND is associated with favorable prognosis and better cancer control. Four decades ago, many urologists did not perform PLND with RC [7]. In 1950, Kerr and Colby noted that the local recurrence rate decreased after cystectomy combined with PLND [8].

In 1973, Dretler et al. [9] reported the value of inclusion of PLND during RC without increasing the morbidity and mortality. Skinner [10] suggested that PLND can cure some patients with metastatic disease, effectively controls pelvic disease and can make a difference in survival.

In 1981 Smith and Whitmore [11] reported on one of the first anatomical LN mapping studies in patients undergoing RC and they suggested the possible therapeutic effect of a systematic bilateral PLND as a major determinant of patient survival. Although there is consensus the PLND should be an integral part of cystectomy; the extension of PLND is not standardized and the number of LNs that should be removed has yet to be defined [12].

In this article we will review the therapeutic and prognostic value of PLND, the optimal surgical template, and the minimum number of nodes to be removed.

## 2. Lymphatic Drainage of the Bladder and Templates of PLND

The lymphatic drainage of the bladder consists of the visceral lymphatic plexus inside the submucosa and the muscular layer, the small intercalated lymph nodes located within the perivesical fat, pelvic collecting trunks which is medial to the iliac LNs, regional pelvic LNs, which include the external and internal iliac, and sacral LNs, lymphatic trunks from the regional pelvic LNs to the common iliac LNs [13]. The pelvic LNs are embedded in fat and difficult to be appreciated during the surgery. The primary drainage sites include external and internal iliac and obturator LNs, secondary drainage from the common iliac LNs, and tertiary drainage from the trigone and posterior bladder wall is to the presacral nodes [14]. LNs mapping studies in RC shows that the rate of positive LNs detected decreased gradually from distal to more proximal sites and the most common site of LN metastasis were in the obturator and iliac LNs. Positive LNs were found in the perivesical fat and in the pelvic region in 22.7% of all patients, in the common iliac nodes in 8%, in the presacral region in 5.1% and at or above the aortic bifurcation in 4% [14]. In another study, the distribution of the LN metastasis in the external iliac, obturator, and internal iliac region was 33%, 38%, and 29%, respectively. Metastases in only one region were found in 33% of patients (13% in the external iliac LNs, 10% in the obturator LNs, and 10% in the internal iliac LNs); 50% of all patients had lymph node metastases in the internal iliac region [15].

Abol-Enein et al. [16] in pathoanatomical study of LN involvement in patients with bladder cancer, concluded that the internal iliac and obtur ator LNs (endopelvic region) is the sentinel region of the lymphatic drainage of bladder cancer and there are no skipped lesions or isolated nodal metastasis above the aortic bifurcation. Thus any metastasis outside the true pelvis occurred only in multinodal disease and it was always associated with involvement of the obturator and/or internal iliac nodes. Others reported that single positive nodes were located outside of the pelvis in 27% of patients [17]. The difference between these finding may be explained by the variation in the natural history of the disease [16].

Early RC series suggested that it was not necessary to include the aortocaval lymph nodes in PLND, as a part of the cystectomy [13, 18]. Others suggested the importance of an extended PLND to include common iliac LNs to remove all potential LN metastases [11].

The limits of each surgical template of PLND are not clearly defined in the urology guidelines. Although the absolute boundaries of the PLND remain a subject of controversy, three categories of PLND are reported in the literature, limited, standard, and extended. The limited PLND drains part of the primary drainage and includes only the obturator and external iliac LNs [7].

The standard PLND was defined as a removal of all nodal tissue of primary and secondary lymphatic drainage of the bladder and encompasses the common iliac bifurcation proximally, the genitor-femoral nerve laterally, the circumflex caudal iliac vein and lymph node of Cloquet distally, and the internal iliac vessels posteriorly, including the obturator fossa. The nodes around the proximal half of the common iliac artery/aortic bifurcation are spared to avoid injury to the hypogastric nerves [19].

The boundaries of extended PLND are 1-2 cm above the aortic bifurcation and common iliac vessels proximally (others may extend the PLND up to the level of inferior mesenteric artery), the genitofemoral nerve laterally, the circumflex iliac vein and lymph node of Cloquet distally, the internal iliac vessels posteriorly, including the obturator fossa, the presciatic nodes bilaterally, and the presacral lymph nodes over the sacral promontory [20].

Leissner et al. [17] defined three different anatomical levels of metastasis: Level I, included in the standard template; Level II, including the aortic bifurcation; Level III, including the para-aortic and paracaval areas. The authors noted that, if there is a nodal metastasis at Level I, positive nodes were also found in 57% and 31% of cases at Levels II and III, respectively. If positive nodes were found at Level II, 35% of cases were positive at Level III. Positive nodes at Level III were found only if metastases were present in 9 or more nodes at Levels I and II.

## 3. Current Practice of PLND

In 2004, Herr et al. [21] reported on 1091 consecutive RC performed by 16 experienced surgeons from 4 institutions between 2000 and 2002. Surgeons performed a standard PLND in 67% of patients, extended PLND in 13% of patients, and for various reasons 20% had a limited (9%) or no node dissection (11%). In analysis of the Surveillance, Epidemiology and End Results (SEER) data of 3603 RC performed between 1992 and 2003, Hollenbeck et al. [22] divided the hospitals according to the node count during cystectomy, low (no patients with ≥ 10 LNs removed), medium (up to 20% of patients with ≥ 10 LNs removed), and high (greater than 20% of patients with ≥ 10 LNs removed). The authors found that only 0–4 nodes were retrieved in 88.9% and 52.8% of cases in the low and high node count hospitals, respectively. The percentages of patients who had ≥ 10 LNs removed were 0% at low LN count hospitals, 12.7% at medium LN count hospitals, and 35.3% at high LN count hospitals. It seems that the majority of cystectomy patients had ≤4 LNs removed irrespective of the hospital and optimal PLND is not commonly performed.

The possible reasons for no or limited PNLD during RC was reported by Koppie et al. [23] who found that patients with older age and higher comorbidities were less likely to have PLND, and when PLND was performed, fewer LNs were evaluated. In another analysis of SEER data, Hellenthal et al. [24] noted that the odds of undergoing PLND (1 or more nodes) decreased nearly 20% per 10-year age increase. Also the odds increased by a factor of 1.5 in the tumor stage TisN0M0 to T3N0M0 and decreased in stage T4N0M0. The same authors concluded that 21% of patients did not have any LNs sampled at radical cystectomy. This number decreased from 37% in 1988 to 16% in 2004. During this period the mean number of LNs removed increased by 2.6 nodes and the percentage of patients undergoing any form of lymph node dissection increased by an average of 19%. As of 2004, 84% of patients had at least 1 LN (and a mean of 13 nodes) examined at cystectomy. Similarly, Koppie et al. [25] noticed that the number of removed LN was associated with year of surgery in their series. The mean number of LNs removed during 1990–1994, 1994–1999, and 2000–2004 was 7.5, 8.6, and 14.7, respectively.

## 4. Therapeutic and Prognostic Value of PLND

### 4.1. Extent and Number of Lymph Nodes Removed

Weingärtner et al. [26] assessed the adequate number of LNs to be removed for achievement of complete and accurate PLND. Standard PLND was performed on 30 human cadavers and 59 consecutive patients with clinically organ confined prostate cancer during radical retropubic prostatectomy. The mean number of LNs removed in the autopsy series and from patients with prostate cancer was 22.7 ± 10.2 and 20.5 ± 6.6, respectively, with many interindividual differences. The authors concluded that the threshold of approximately 20 nodes was sufficient. The average number of nodes removed during the standard and extended PLND was reported to be 13 (9–18) and 31.5 (14.7–50), respectively [7].

The number of LNs retrieved during PLND is quite variable and different cutoff number of LNs that should be dissected was suggested. So far there is no consensus available for the standard number of LNs that should be retrieved in patients with bladder cancer. The higher number of LNs retrieved may reflect more complete RC and PLND. Several studies showed that disease-free survival or recurrence-free survival improved with more number of nodes retrieved which was an independent prognostic factor even after adjusting for node status, surgical margin, and pT stage [27–29]. Herr et al. [27] found that the survival rate in the node negative patients was improved with more number of nodes removed, which may be attributed to an improved staging, and possible removal of undetectable micrometastasis. The authors suggested PLND of at least 9 or more as a minimum standard provides individual prognostic information.

Others reported on decreased risk of death in patients who had 10–14 lymph nodes removed. Patients with less than 3 LNs retrieved were at significantly higher risk of death from bladder cancer than those with greater than 3 [30]. Leissner et al. [31] found that the extended PLND of ≥ 16 LNs correlated with a higher percentage of patients with documented nodal metastases. There was a significant correlation between the number of removed LNs and the tumor-free 5-year survival in patients with pT1, pT2, or pT3 tumors.

Fang et al. [32] reported on 349 patients who underwent RC and PLND between March 2000 and February 2008. The authors established an institutional policy mandating at least 16 LNs be examined in March 2004. Of all, 147 and 202 patients underwent surgery before and after the policy was implemented, respectively. The median number of LNs examined increased from 15 in the period before policy implementation to 20 in the 4 years after. Survival rates increased from 41.5% in the 4 years before policy implementation to 72.3% in the 4 years after. Capitanio et al. [33] reported on a multicenter study to identify the probability of finding one or more positive LNs based on the number of LNs removed. The authors found that removing 45 LNs yielded a 90% probability. However, removing either 15 or 25 LNs indicated probability of 50% and 75%, respectively. They concluded that removing 25 LNs might represent the lowest threshold for the extent of PLND at RC. Others reported that at least 23 nodes would need to be removed in order to identify 80% of positive nodes [34].

The number of LNs resected is a surrogate for the extent of dissection and the quality of RC. Thus it seems to be difficult to establish a minimum or threshold number of LNs that should be removed during PLND due to the lack of a standardized template of PLND. According to Koppie et al. [25] removing 10 LNs may represent a thorough LN cleanout from a limited LN template, or a relatively incomplete dissection of LNs from an extended LN template. The authors concluded that no evidence was found to support a minimum number of LNs sufficient for optimizing bladder cancer outcomes when a limited or extended PLND is performed during RC. The probability of survival continues to increase as the number of LNs retrieved increases. Also, the authors recommended more extended PLND at the time of RC.

Several studies demonstrated that extended PLND decrease local recurrence and improve cure rates when it compared to limited and standard PLND [31, 35]. Dhar et al. [36] reported on a multicenter study comparing the recurrence patterns and survival of 658 patients who underwent RC with either limited or extended PLND. The overall LN-positive rate was 13% and 26% for patients with limited and extended PLND, respectively. The 5-year recurrence-free survival was 77% for pT2N0, 57% for pT3N0, and 35% for node-positive tumors in the extended PLND group versus 67%, 23%, and 7%, respectively, in the limited PLND group ( *P* < 0.0001).

In another study, LN metastases were detected in 38% and 17% of the extended and limited dissection groups, respectively. There was no significant difference in survival or time to recurrence between the 2 groups. However, the multivariate analysis demonstrated significantly improved survival and time to recurrence in the patients with extended PLND [37].

In addition to its therapeutic benefit, extended PLND offers more accurate staging compared to a limited/standard PLND. Dangle et al. [34] found that limited PLND would have missed 25% of LN-positive patients whereas standard PLND would have missed 11% of LN positive cases. Others noted that limited and standard PLND would have missed 27 and 10% of LN metastases in patients with a single positive lymph node, respectively [17]. Seiler et al. [15] found that PLND that do not include the internal iliac region misses 26% of all pelvic lymph nodes, 29% of metastases, and understages a substantial number of patients as pN0 (10%).

Although surgical cure is rare in patients with gross nodal metastasis (N2-3), the RC with extended PLND can provide cure in this group of patients. The 10-year disease-free survival was reported in 24% of patients with surgery alone and 76% of patients died of disease. Thirty-two percent of patients with T2 tumors survived versus 9.7% of patients with stage T3 tumors [38].

### 4.2. Tumor Burden

The number of positive nodes retrieved is indicative of the tumor burden and considered as independent from the number of nodes removed. The survival rate is directly correlated with number of LN metastasis. Several cut off number of positive LNs was reported. One of these studies showed that the survival of patients with positive nodes was significantly better if ≤4 positive nodes were removed than if there were >4 positive nodes (37% versus 13%) [27]. Improved overall survival was shown in a study by Lerner et al. [39] when 5 or fewer positive nodes were detected (40% versus 10%). The mean 3-year survival for patients with 1, 2–5, and > 5 positive LNs was 58.6%, 31.8%, and 6.8%, respectively [6]. Fleischmann et al. [40] found that the Overall 5-year survival was 35% and 17% in patients with <6 and ≥6 positive LN, respectively. Others showed that the 10-year recurrence-free survival was significantly better in patients with ≤8 positive nodes than in those with >8 metastatic nodes (40% versus 10%) [41]. Kassouf et al. [42] reported that the number of positive nodes was significantly associated with recurrence-free survival on univariate analysis (*P* = 0.04), but lost statistical significance on multivariable model (*P* = 0.055).

### 4.3. Lymph Node Density

LN density defined as number of positive LN divided by the total number of nodes removed and examined. It included 2 prognostic factors the tumor burden and the extent of PLND. The most commonly utilized LN density cut-point was 20%. The concept of LN density was introduced and named “ratio-based” lymph node staging by Herr in 2003 [43]. They found that the 5-year overall survival decreased from 64% when the ratio was ≤20% down to 8% when it was >20%. The same concept was later reported under a different name (LN density) by Stein et al. [41] who showed decreased 10-year recurrence-free survival from 43% when the LN density was ≤20% to 17% when it was >20%. Others reported that the LN density showed some predictive ability, especially at a cutoff of 50% [30].

Kassouf et al. [44] compared nodal status and LN density in a multivariate model. For powerful LN density, a minimum number of 9 nodes need to be resected. The authors found that only LN density >20% predicted decreased disease-specific survival and remained prognostic in patients who received adjuvant chemotherapy. Also the LN density is superior to tumor-node-metastasis (TNM) classification for nodal status in predicting disease-specific for susvii patients with LN-positive disease. This study and others support the use of LN density in the pathologic staging of node-positive bladder cancer [45].

Although Abdel-Latif et al. [6] found that both number of positive nodes (1 versus 2–5 versus >5) and LN density (<10 versus 10–20 versus >20%) showed statistical significance on univariate analyses, only the number of positive nodes remained significant on multivariate modeling. Wright et al. [28] found statistically significant correlation between the number of positive nodes (1 versus 2 versus 3 versus >3) and lymph node density, and disease-specific and overall survival.

### 4.4. Lymphovascular Invasion

Lymphovascular invasion (LVI) means the presence of tumor cells in the endothelium-lined space. In a recent longitudinal evaluation of the prognostic value of LVI, Resnick et al. [46] found that 12.3% of patients had LVI at transurethral resection of the bladder tumour (TURBT) compared to 33.1% at RC. The risk of nodal disease was higher in those patients with LVI at TURBT than in those with no evidence of LVI at TURBT (48.3% versus 25.0%, *P* < 0.001). The authors concluded that the LVI has a useful prognostic value and should be incorporated into clinical decision making, particularly for RC in patients with superficial bladder carcinoma and the need for neoadjuvant chemotherapy.

Quek et al. [47] noted that the LVI is an important and independent prognostic variable in patients with invasive bladder cancer. It was significantly correlated with positive surgical margins, high pathological stages, older patients, and sex (female). Ten-year recurrence-free survival in patients without LVI was 74% compared with 42% in those with LVI (*P* < 0.0001). Similarly 10-year overall survival was 43% in patients without LVI compared with 18% in those with LVI (*P* < 0.0001).

In another study, LVI was not significantly associated with age or sex, but was significantly associated with high pathological grade (*P* = 0.028), stage (*P* < 0.001), and node metastasis (*P* < 0.001). At the multivariate analysis, LVI was an independently significant prognostic factor for disease-specific survival [48, 49]. Others noted that the LVI in node-negative patients is an adverse prognostic factor on univariate analysis of disease-specific survival, but not an independent prognostic factor on multivariate analysis [50].

Shariat et al. [51] reported on international validation of the prognostic value of LVI in 4257 patients treated with RC. In analysis, LVI was associated with both disease recurrence (hazard ratio 1.43, *P* < 0.001) and cancer-specific mortality (1.45, *P* < 0.001). In patients with negative LNs, LVI was independently associated with and improved the predictive accuracy of the standard predictors for recurrence (hazard ratio 1.68, *P* < 0.001; +2.3%) and cancer-specific mortality (1.70, *P* < 0.001; +2.4%). The authors concluded that the LVI should be included in the staging of bladder cancer.

### 4.5. Extracapsular Extension of the Lymph Node (ECE)

Currently, there are a few reports in the literature investigating the prognostic value of ECE of LN metastasis. Perforation and extension of the tumor growth outside the LN capsule indicate aggressive behavior of the tumor. Fleischmann et al. [40] found the ECE was observed in 58% of patients and in the multivariate analysis for recurrence-free survival, ECE of LN metastases was the strongest prognostic factor (*P* = .019) of recurrence-free and overall survival. In contrast, Kassouf et al. [52] suggests that ECE is not an independent prognostic factor for overall survival, disease-specific survival, and recurrence-free survival in patients with positive LNs.

### 4.6. The Aggregate LN Metastasis Diameter (ALNMD)

Some studies suggested that the size of the largest LN metastasis and/or the aggregate LN metastasis diameter (ALNMD) may provide prognostic information about the extent of LN metastasis, and the patients' survival. Mills et al. [53] noted that there is a significant association between the diameter of the largest LN metastasis and overall survival. Very recently, Stephenson et al. [54] reported on 134 positive LN patients treated with RC and minimum standard PLND. The median overall survival was 26 months for patients with ALNMD ≤20 mm versus 11 months for those with ALNMD >20 mm (*P* = .001). The authors concluded that ALNMD is a significant predictor of recurrence-free survival and overall survival and may provide a useful parameter to be included in the TNM-staging systems.

## 5. Role of the Pathologist in LNs Assessment

The accurate assessment of LN specimen depends on the carful work of the pathologist when searching the specimen for LNs and the way of specimen submission for pathological examination. Bochner et al. [55] found that individual LN specimen yielded more LNs compared to en bloc specimen in standard PLND (8.5 versus 2.4 LNs, *P* = 0.003) and extended PLND (36.5 versus 22.6 LNs, *P* = 0.02). This result confirmed by Stein et al. [56] who suggests 13 separate nodal packets to increase the total number of lymph nodes removed compared with en bloc submission.

The traditional method of detecting the LNs by sectioning and palpating the specimen may fail to detect the very small LNs. Koren et al. [57] described a new Lymph-node revealing solution (LNRS) for detecting LNs in PLND specimen. The solution comprised 95% ethanol, diethyl ether, glacial acetic acid, and buffered formalin and used to degrease the tissue. The authors found that using the LNRS doubled the number of LNs yield, detected significantly smaller LNs, and improved nodal staging.

Herr et el. [58] reporting on pathologic evaluation of RC specimens, found that in 18% of patients, pathologists did not mention either the presence or the number of LNs. Standardized pathologic evaluation and reporting of RC and LN specimens is critical in cancer staging and design of clinical trials.

## 6. Laparoscopic/Robot-Assisted PLND

Laparoscopic PLND for prostate cancer was initially described by Schuessler et al. [59]. The laparoscopic surgery is minimally invasive with advantages of decreased blood loss, shorter hospital stay, and early recovery. Several reports showed that there is no significant difference in the intraoperative complications and the number of LNs removed by laparoscopic approach when compared with open surgery [60, 61].

Introduction of robot-assisted laparoscopic surgery added more field magnification with 3-dimensional vision and simulates the movements of the surgeon's wrist. Guru et al. [62] evaluated the number of LNs yield during robot-assisted RC and found that the mean operative time for PLND is 44 minutes (19–85) and the mean number of LNs removed was 18 (6–43).

It seems likely that the robot-assisted PLND can produce comparable results to open surgery; however, more studies of the techniques and learning curve are still needed [63].

## 7. Morbidity and Mortality of PLND

Although, RC is major surgery with potential high rates of complications, extended PLND does not increas morbidity or mortality. There is no significant difference between LN-positive and -negative patients in terms of postoperative complications [41]. In a study comparing extended PLND (up to the aortic bifurcation) to a more limited PLND, similar mortality rates were observed in the 2 groups [35]. Similarly, Leissner et al. [31] observed that the postoperative complications such as lymphocele and lymphoedema were similar in patients with <16 lymph nodes removed and patients with >16 nodes removed (2% versus1%). Although the extended PLND increased the operative duration by 63 minutes, the limited and extended PLND patients did not differ significantly in terms of perioperative mortality and morbidity. Complications requiring surgical interventions occurred in 9% patients in limited PLND and 11% in extended PLND group (*P* = 0.28) [64].

## 8. Conclusion

PLND is an essential part of the surgical treatment of bladder cancer for its staging, curative, and prognostic role. The benefits of extended PLND were demonstrated in several studies with no significant difference in morbidity and mortality when compared to standard PLND. Despite the growing evidence that support the extended PLND up to the inferior mesenteric artery, the optimum PLND template is still controversial and its boundaries and the number of retrieved LNs have not yet been defined. Well-designed randomized controlled trials comparing standard to extended PLND in RC patients is still needed. The extent of PLND and the number of positive LNs are well-established risk factors; however, the cut off number of positive LNs is still to be defined. Several reports suggested that LVI and LN density should be included in the pathologic staging of bladder cancer. The prognostic value of ECE and ALNMD still need more investigations.
